# Revision in the first steps of the biosynthesis of the red antibiotic prodigiosin: use of a synthetic thioester to validate a new intermediate†

**DOI:** 10.1039/d0cb00173b

**Published:** 2021-01-15

**Authors:** Maxime Couturier, Hiral D. Bhalara, Rita E. Monson, George P. C. Salmond, Finian J. Leeper

**Affiliations:** Yusuf Hamied Dept. of Chemistry, University of Cambridge Lensfield Road Cambridge CB2 1EW UK fjl1@cam.ac.uk; Dept. of Biochemistry, University of Cambridge Tennis Court Road Cambridge CB2 1QW UK

## Abstract

A biosynthetic pathway for the red-antibiotic, prodigiosin, was proposed over a decade ago but not all the suggested intermediates could be detected experimentally. Here we show that a thioester that was not originally included in the pathway is an intermediate. In addition, the enzyme PigE was originally described as a transaminase but we present evidence that it also catalyses the reduction of the thioester intermediate to its aldehyde substrate.

## Abstract 2

A revision is proposed to the biosynthetic pathway to the well-known red pigment prodigiosin *via* a new thioester intermediate.

## Graphical abstract

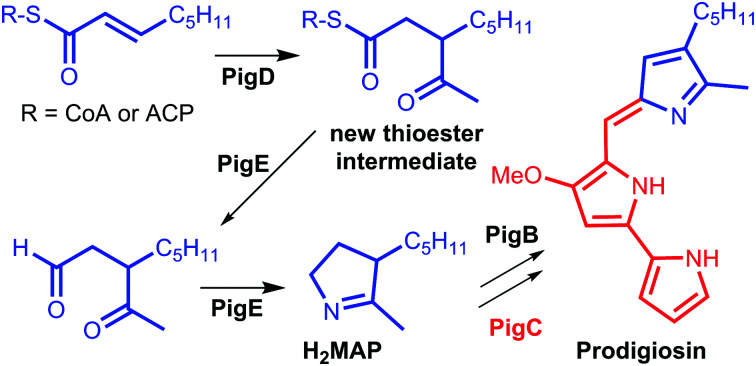

## Introduction

Prodigiosin **11** is a tripyrrolic natural product produced by a wide range of bacteria.^1^ It originally caught researchers’ attention because of its bright red colour, but its various biological activities have renewed interest in this molecule in recent decades.^2^

**Fig. 1:**
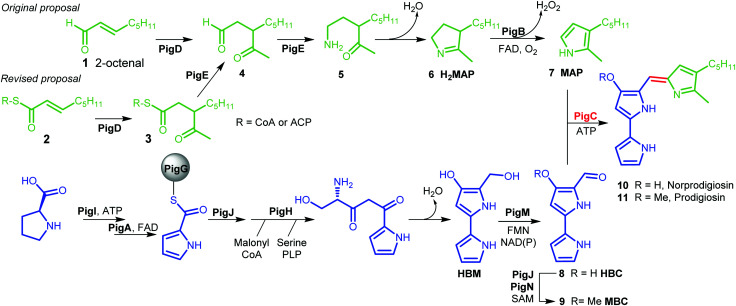
Biosynthesis of prodigiosin; green; formation of MAP, blue: formation of MBC, red: condensation. HBM is 4-hydroxy-2,2′-bipyrrole-5-methanol and HBC is 4-hydroxy-2,2′-bipyrrole-5-carbaldehyde.

The biosynthetic pathway to prodigiosin was established by Williamson *et al. via* cross-feeding experiments of various knock-out mutants of *Serratia* ATCC sp.39006 (*S*39006).^3^ They showed that prodigiosin resulted from a bifurcated pathway (Fig. 1) with the independent synthesis of monopyrrole 2-methyl-3-amylpyrrole (MAP) **7** and bipyrrole 4-methoxy-2,2′-bipyrrole-5-carbaldehyde (MBC) **9** before a final condensation step catalysed by the enzyme PigC.

MBC is an intermediate in the biosynthesis of other prodiginines, natural products which share the tripyrrolic structure of prodigiosin **11**. In particular, the enzymes involved in the biosynthesis of MBC in *Streptomyces coelicolor A(3)* have been well characterised.^4^ The biosynthesis of MAP, on the other hand, is specific to the formation of prodigiosin **11**. 2-Octenal **1** was originally proposed to be the starting point of the pathway.^3^ Addition of an acetyl group from pyruvate by PigD was proposed to form 3-acetyloctanal **4**, which then underwent a transamination catalysed by PigE,^5^ leading to amino ketone **5** which spontaneously cyclises to form dihydroMAP (H_2_MAP) **6**. H_2_MAP is then oxidised to MAP **7** by PigB.^6^

However, Dresen *et al.* showed that, when presented with an aldehyde such as 2-octenal **1**, PigD catalysed the direct addition of the acetyl group onto the aldehyde carbon, whereas, with less reactive α,β-unsaturated ketones and thioesters, attack on the β position was preferred.^7^ Kasparyan *et al.* extended this work to some homologues of PigD and suggested that thioester **3** may be the true natural product of PigD.^8^

In this work, we show that thioester **3** is indeed an intermediate in the MAP biosynthetic pathway. In addition, we provide evidence that PigE catalyses the reduction of the thioester intermediate to aldehyde **4** as well as catalysing the transamination of that aldehyde. As a result, we propose an alternative biosynthetic pathway for the formation of MAP which uses 2-octenoyl thioester **2**, either with coenzyme A (CoA) or an acyl carrier protein (ACP), as its starting point (Fig. 1).

## Results and discussion

### Preparation of potential substrates

**Fig. 2:**
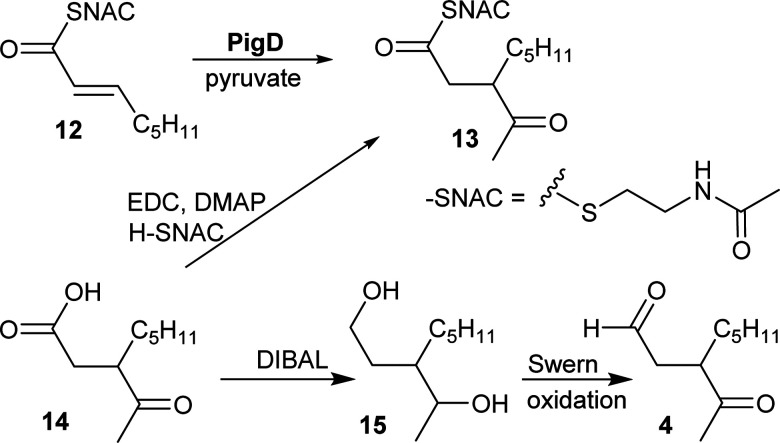
Syntheses of potential substrates of PigE.

CoA and ACP thioesters are common intermediates in biosynthesis. In particular, straight-chain thioesters with even numbers of carbons are readily available from fatty acid biosynthesis (or degradation). In addition, it has been shown that PigD catalyses the transfer of an acetyl group from pyruvate to *N*-acetylcysteamine (NAC) thioester **12** to give thioester **13** (Fig. 2).^7,8^ Considering that NAC thioesters mimic CoA or ACP thioesters, we synthesised **13** to investigate if it could be converted to prodigiosin by *S*39006.

As thioesters are prone to hydrolysis and all testing of **13** would be done in aqueous solution, its hydrolysis product, 3-acetyloctanoic acid **14**, was also synthesised, as a negative control, and the known PigE substrate 3-acetyloctanal **4** was synthesised as the positive control.

Acid **14** was obtained in three steps from 2-octanone (see ESI†): bromination on C-3 was followed by displacement of bromide by dimethyl malonate and then hydrolysis/decarboxylation. Acid **14** was then converted to **13***via* thioesterification with *N*-acetylcysteamine. Aldehyde **4** was obtained by reduction of **14** to the diol **15** followed by Swern oxidation.

### Chemical complementation assays

*S*39006 *ΔpigD* has an in-frame deletion in *pigD*, avoiding any downstream polarity in the prodigiosin biosynthetic operon, and presents a white phenotype.^3^ However, when fed with an intermediate accepted by one of the subsequent enzymes in the MAP biosynthetic pathway this strain produces **11** and therefore presents a red phenotype.^3,6^

**Fig. 3:**
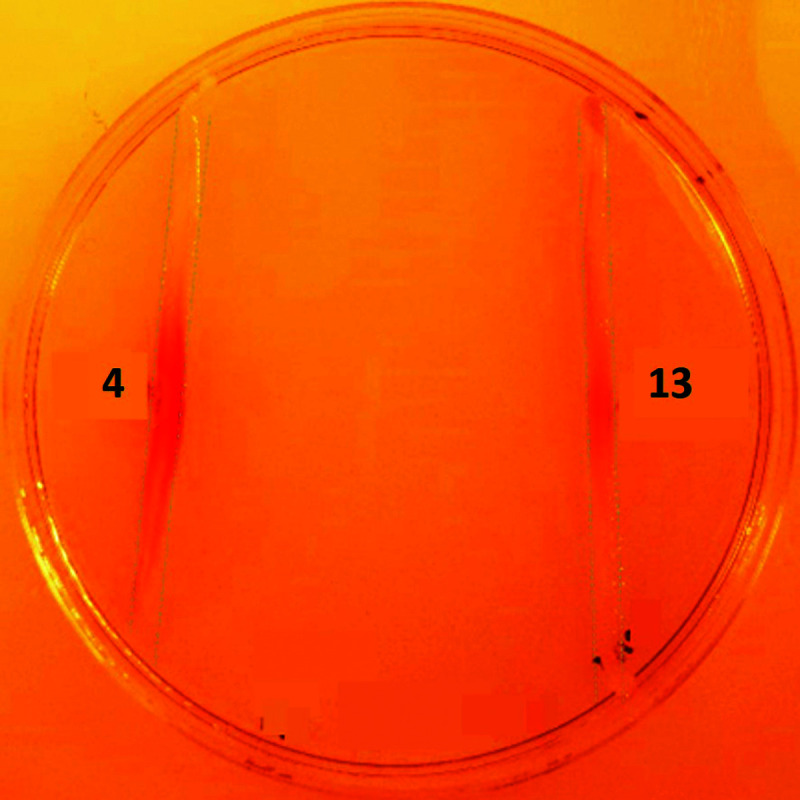
Chemical complementation of *S*39006 *ΔpigD* with aldehyde **4** (left) and thioester **13** (right).

In a qualitative assay, *S*39006 *ΔpigD* was streaked on agar plates and spots (2 μl, 1 M) of aldehyde **4** and thioester **13** were placed next to the streaks. After 12 h, a red colour was visible in the streak next to the spot in both cases (Fig. 3), suggesting that both compounds could restore prodigiosin production. The colour was less intense in the case of the thioester, perhaps suggesting less efficient uptake into the cell or less efficient utilisation of the NAC thioester than the true thioester intermediate.

**Fig. 4:**
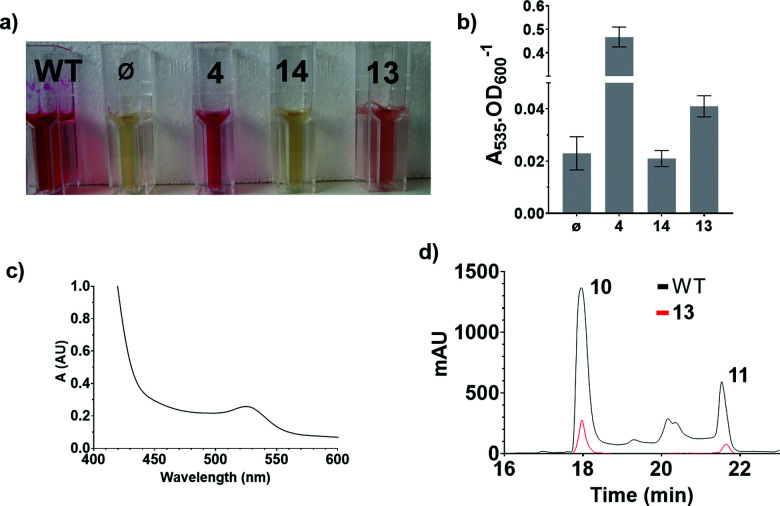
Results of the quantitative chemical complementation assays; (a) ethanolic extract of cultures of *S*39006 WT and *S*39006 *ΔpigD* incubated with no additive (*ϕ*), aldehyde **4**, carboxylic acid **14** or thioester **13**; (b) Absorbance at 535 nm normalised by OD_600_ for the specified extracts; (c) UV spectrum of the extract of *S*39006 *ΔpigD* inoculated with thioester **13**; (d) Comparison of the HPLC traces (535 nm) of the extracts of *S*39006 WT and *S*39006 *ΔpigD* incubated with thioester **13**.

These results were confirmed by a quantitative complementation assay in liquid culture. After optimisation to overcome low levels of production of **11** due to the toxicity of **4**, *S*39006 *ΔpigD* was treated with **4**, **13** or **14** (1 mM) for 16 h and prodigiosin was then extracted from cell pellets with acidified EtOH and quantified by its absorbance at 535 nm.^9^ Again the results (Fig. 4) were that pigmentation was observed with aldehyde **4** and thioester **13** but not with carboxylic acid **14**. The UV spectrum of the extract of the culture treated with **13** showed a maximum at 535 nm (Fig. 4c), consistent with prodigiosin.

Extracts from cultures of *S*39006 *ΔpigD* not treated with any substrate have some absorption at 535 nm but this is a sloping baseline and no peak is seen. Nevertheless, to confirm that the increase in absorbance during the chemical complementation experiments was due to prodigiosin and not some other metabolite, an ethanolic extract from a culture of wild-type *S*39006 was analysed by HPLC. Two peaks absorbing at 535 nm were observed and LCMS (see ESI†) showed they were prodigiosin **11** and norprodigiosin **10**. Norprodigiosin results from the condensation of the unmethylated bipyrrole HBC **8** with MAP **7** (see Fig. 1). The peaks corresponding to **11** and **10** were also observed in the HPLC trace of the extract from *S*39006 *ΔPigD* treated with **13** (Fig. 4d). This proves that thioester **13** can restore production of prodigiosin in *S*39006 *ΔpigD*. Thus it is very likely that a related thioester is an intermediate in the biosynthesis of MAP.

### Bioinformatic analysis of PigE

The presence of a thioester intermediate in MAP biosynthesis implies that a reduction step converting the thioester to an aldehyde needs to be added to the original pathway. The possibility that PigE had thioester reductase activity as well as the established aminotransferase activity was investigated.

The sequence of PigE was analysed using the NCBI BLAST software.^10^ This showed that the C-terminal region of PigE (residues 394 to 848) had strong similarity with pyridoxal phosphate (PLP)-dependent aminotransferases, particularly class III aminotransferases that generally act on or produce terminal alkylamines.^11^ A crystal structure of the C-terminal domain with PLP bound has been published.^5^ The N-terminal region (residues 2 to 341) showed homology with acyl-ACP reductases, which catalyse the reduction of fatty acyl ACP thioesters to the corresponding aldehydes.^12^ The majority of proteins that showed good similarity with full length PigE were homologues in other organisms known to produce prodigiosin or related compounds (*e.g.* TamH in tambjamine biosynthesis^13^).

PHYRE2 homology modelling software^14^ generated a structural model for the N-terminal domain (see ESI†) using the recently published structure of the acyl-ACP reductase (AAR) involved in cyanobacterial biosynthesis of hydrocarbons (26% identity).^15^ The second best hit was glutamyl tRNA reductase (GluTR) from *Methanopyrus kandleri* (17% identity), which catalyses the transformation of glutamyl tRNA into glutamate-1-semialdehyde, the first committed step of heme biosynthesis in plants, algae and most bacteria. Both these enzymes initially transfer the acyl group onto an active site cysteine residue and it is this thioester intermediate that is subsequently reduced by NADPH.^16^ This cysteine residue is conserved in PigE (Cys296) and is in the same position in the homology model as Cys294 in AAR when the two structures are overlaid. Interestingly both AAR and GluTR have been shown to channel their product aldehydes directly to the next enzyme in the pathway *via* an exit tunnel that is distinct from the substrate entry route. For AAR the next enzyme is a di-iron-dependent aldehyde-deformylating oxygenase (ADO),^15^ but for GluTR it is a PLP-dependent aminotransferase, glutamate-1-semialdehyde aminomutase (GSAM).^17^ It seems likely, therefore, that the reductase and aminotransferase domains of PigE form a structure similar to the GluTR-GSAM complex and substrate channelling of aldehyde **4** may occur.

The bioinformatic analysis, therefore, reveals the presence of two domains in PigE: a C-terminal aminotransferase domain, and a N-terminal thioester reductase domain. This strongly suggests that the N-terminal domain of PigE is the protein that catalyses the reduction of the thioester intermediate.

### *In vitro* characterisation of PigE

**Fig. 5:**
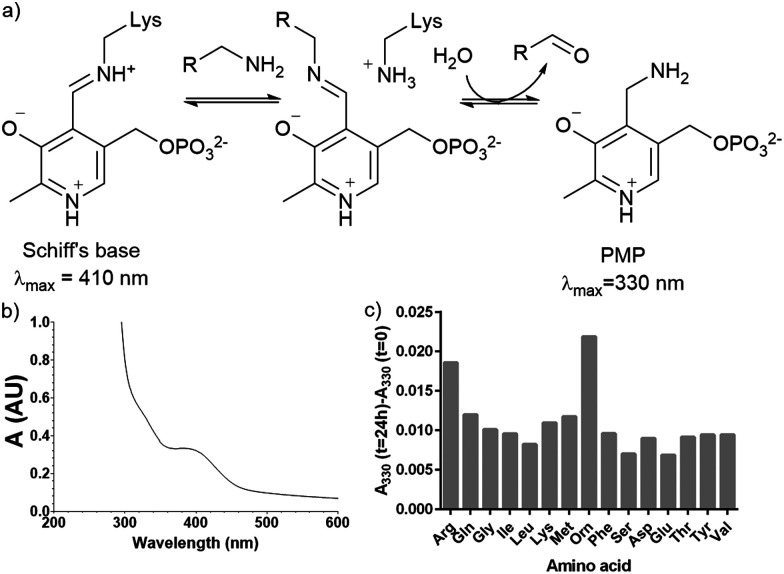
(a) Intermediates in PLP-catalysed transamination; (b) UV spectra of isolated PigE; (c) change in the *A*_330_ of PigE after 24 h in the presence of various amino acids.

Plasmid pQE80-L*::oriT* containing *pigE* from *S*39006 was transformed into *E*. coli BL21, giving strain *E. coli* pPigE. PigE protein was purified from cultures of this strain. A UV spectrum of the purified PigE showed a peak at 410 nm, consistent with PLP bound as its Schiff's base with the active-site lysine residue. (Fig. 5).

To determine the likely amino donor in the transaminase reaction PigE was incubated with various amino acids individually and the formation of the pyridoxamine phosphate (PMP) intermediate was monitored by recording the absorbance at 330 nm. The most significant increase was observed with ornithine and arginine (Fig. 5c) suggesting that PigE is a transaminase dependent on these amino acids.

**Fig. 6:**
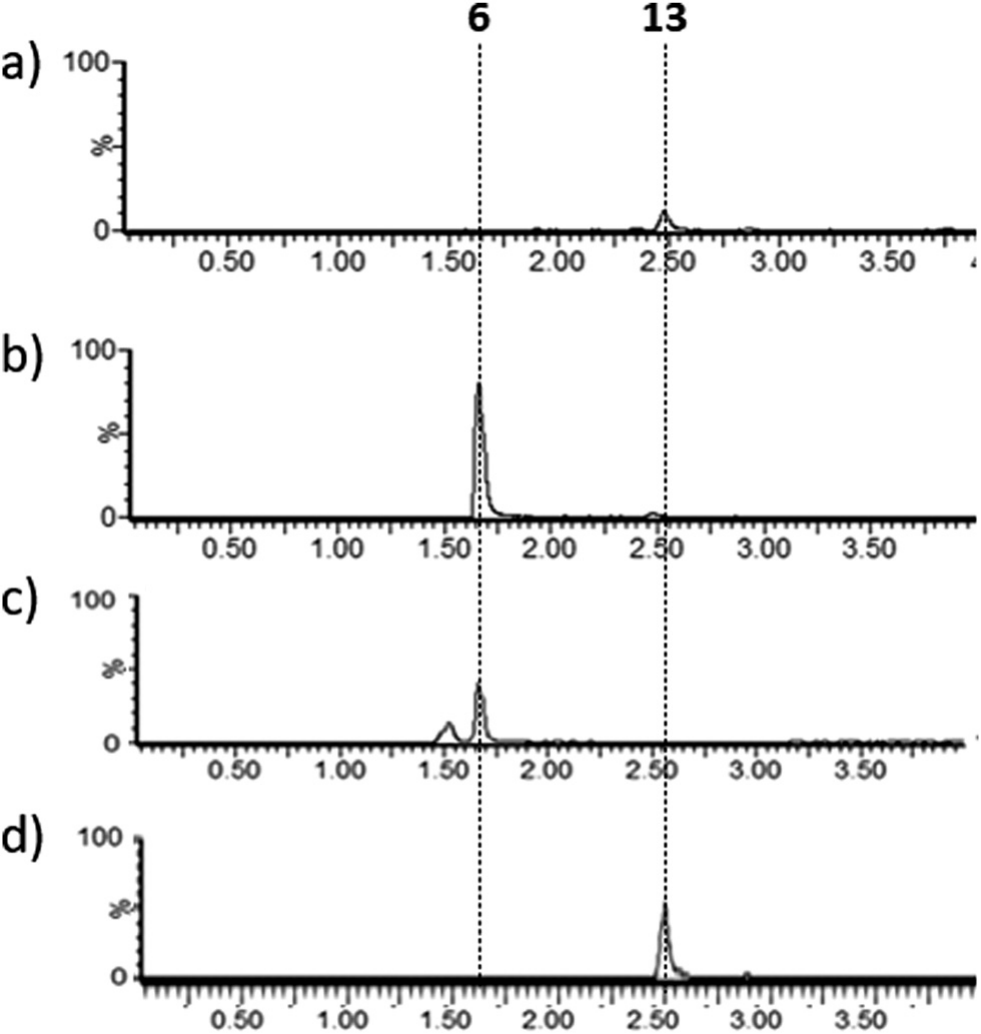
Extracted ion chromatograms (*m*/*z* 154.2 + 288.2) (a) and (b) after incubation of **13** with cell lysates of (a) *E. coli* BL21 or (b) *E. coli* pPigE, (c) synthetic H_2_MAP **6** (*m*/*z* 154.16, **6** + H^+^), (d) synthetic **13** (*m*/*z* 288.16, **13** + H^+^). Retention times corresponding to **6** and **13** are shown.

Lysates prepared from *E. coli* BL21 and *E. coli* pPigE were supplemented with thioester **13**, ornithine and PLP and incubated at 30 °C for 16 h. After removing cell debris, the resulting mixtures were analysed by LCMS and compared with synthetic samples of H_2_MAP **6** and thioester **13** (Fig. 6). In the extract from *E. coli* BL21, only the peak corresponding to the thioester **13** was visible. In the extract from *E. coli* pPigE, the main peak corresponded to H_2_MAP **6** and only a very small peak corresponding to **13** was visible. This is a strong indication that the presence of PigE was necessary for **13** to be reduced in addition to the transamination to give **6**.

When purified PigE was incubated with thioester **13**, ornithine and NAD(P)H at 30 °C for 16 h in phosphate buffer, no peak corresponding to H_2_MAP could be detected by LCMS. Similarly experiments with the same substrates in the presence of a variety of metal ions failed to show any significant change in absorbance of NAD(P)H at 340 nm over time resulting from the presence of PigE. Taken together these experiments suggest that additional cofactors and/or metal ions contained in the cell lysates may be necessary for rapid reaction to occur. It is noteworthy that the activity of GluTR, to which the N-terminal domain of PigE may be related, is affected by several regulatory proteins and by the presence of the tRNA^18^ and activity can easily be lost by oxidation of the active site cysteine.^16^

## Conclusions

We have shown that thioester **13** can restore production of prodigiosin in the deficient strain, *S*39006 *ΔpigD*. In the presence of PigE, the thioester can be reduced, and the resulting aldehyde undergoes transamination, leading to the next biosynthetic intermediate, H_2_MAP.

We therefore propose the revised biosynthetic pathway for prodigiosin shown in Fig. 1. Our results, along with the bioinformatic analysis, support the notion that PigE is a bifunctional enzyme, with a thioester reductase domain in its N-terminal region and a transaminase domain in its C-terminal region. We also propose that the amino donor for the transaminase activity is mostly likely ornithine or arginine.

## Experimental

### Prodigiosin production and quantification

#### Prodigiosin production from S30096 WT

Luria-Bertani broth (LB: tryptone 10 g L^−1^, yeast extract 5 g L^−1^, NaCl 10 g L^−1^) supplemented with 0.25 M sorbitol was inoculated with an overnight culture of the strain. The culture was incubated at 30 °C, with shaking at 250 rpm for 16 h. 25 ml of cells were then pelleted (2219 g, 20 min, 4 °C) and the supernatant was discarded. The pellets were vortexed for 1 min in 5 ml of acidified EtOH (4% 1 M HCl). After centrifugation the supernatant gave the desired prodiginine extract.

#### Prodigiosin production from S39006 ΔpigD

The strain was grown in LB supplemented with 0.25 M sorbitol at 30 °C, with shaking at 250 rpm until the OD_600_ reached 4. Then **4**, **13** or **14** (1 mM final concentration) was added and the incubation extended for a further 16 h. Cells were then pelleted and prodiginine extracted as described above.

#### Prodigiosin quantification

A direct measurement of the absorbance at 535 nm was performed on a Cary 300-Bio UV-visible spectrophotometer.

### Cell extract assays

*E. coli* BL21 or *E. coli* pPigE were cultured in 200 ml of LB until and OD_600_ = 0.6. Cultures were then induced with 1 mM IPTG at 16 °C for 14–16 h. Cells were harvested by centrifugation at 4500 rpm for 20 min at 4 °C. The cells were held on ice for 15 min and then resuspended in 20 ml of phosphate buffer. After a further 30 min on ice, the cell suspensions were sonicated (6 × 20 s with 30 s breaks). Eppendorf tubes were charged with **13** (1 mM), and ornithine and PLP (1.25 mM final concentrations), then cell lysate was added to a total volume of 0.5 ml. The resulting mixture was gently shaken overnight at 30 °C, then centrifuged and the supernatant was analysed directly by LCMS.

## Conflicts of interest

There are no conflicts to declare.

## Supplementary Material

CB-002-D0CB00173B-s001
