# Supplementary material for: Revision in the first steps of the biosynthesis of the red antibiotic prodigiosin: use of a synthetic thioester to validate a new intermediate
Source: RSC Chem Biol. 2021 Jan 15;2(2):551–5. doi: 10.1039/d0cb00173b (PMC8341465; doi:10.1039/d0cb00173b)

Electronic Supplementary Material (ESI) for RSC Chemical Biology.
This journal is © The Royal Society of Chemistry 2021

# Revision in the first steps of the biosynthesis of the red antibiotic prodigiosin: use of a synthetic thioester to validate a new intermediate

Maxime Couturier,[a] Hiral D. Bhalara,[a] Rita Monson,[b] George P. C. Salmond,[b] and Finian J. Leeper*[a]

[a] Department of Chemistry, University of Cambridge, Lensfield Road, Cambridge CB2 1EW, U.K.

[b] Department of Biochemistry, University of Cambridge, Tennis Court Road, Cambridge CB2 1QW, U.K.

## SUPPORTING INFORMATION

### General considerations

Standard solution-phase chemistry was used for the synthesis of the various biosynthetic intermediates and their analogues. All reactions were carried out under $N_2$ in pre-dried glassware and all organic solvents used were freshly distilled. Solvents and reagents for anhydrous reactions were dried by conventional methods prior to use. Milli-Q deionised water was used in all chemical reactions and biochemical work. High temperature reactions were carried out using a silicone oil bath. Yields refer to chromatographically and spectroscopically pure compounds. Microanalyses were performed by the University of Cambridge Microanalytical Laboratory in the Department of Chemistry and are quoted to the nearest 0.1% for all elements except for hydrogen, which is quoted to the nearest 0.05%. Reported atomic percentages are within the error limits of ± 0.3%.

#### Nuclear Magnetic Resonance spectroscopy

NMR Spectra were recorded in deuterated solvents (as specified) using a Bruker AM/DPX-400 ($^1$H NMR at 400 MHz, $^{13}$C NMR at 100 MHz). Chemical Shifts (δ) are quoted in parts per million (ppm) and referenced to solvent peaks. Coupling constants (*J*) are reported in Hz and are rounded to the nearest integer. The multiplicities and general assignments of the spectroscopic data are denoted as: singlet (s), doublet (d), triplet (t), quartet (q), quintet (quin), unresolved multiplet (m), and broad (br).

#### LC-MS

LC-MS analyses were performed with an Waters H-Class UPLC on a Waters Acquity UPLC BEH C18, 1.7 µm column eluted with a gradient of 10 mM aqueous ammonium acetate containing 0.1% formic acid to 95% acetonitrile over 3 min, coupled to a Waters Micromass ZQ Quadrupole Mass Analyser using electrospray ionisation (ESI) with accuracy no greater than 0.4 Da.

#### Mass Spectrometry

Accurate masses were obtained with a Waters LCT Premier high-resolution mass spectrometer and/or a Waters Xevo G2-S.

#### Infra-Red (IR) Spectroscopy

IR spectra were recorded neat on a diamond/ZeSe plate using a Perkin-Elmer Spectrum One FT-IR Universal Attenuated Transmittance Reflectance (ATR) sampling accessory spectrometer with internal referencing. Characteristic absorption maxima ($\lambda_{max}$) are reported in wavenumbers (cm$^{-1}$) and the following abbreviations are used: w, weak; m, medium; s, strong; br, broad.

### Silica Gel Chromatography

Flash Column chromatography was performed using 230-400 mesh Kieselgel 60 silica. Analytical thin layer chromatography (TLC) was performed on commercial silica gel plates (Merck glass- or aluminium-backed plates coated with a 0.20 mm layer of silica gel 60 with fluorescent indicator UV254). These plates were visualised using either ultraviolet light (254 or 365 nm), or by staining the plates with potassium permanganate or vanillin solutions or with Ehrlich's reagent.

### Ultraviolet-Visible (UV-Vis) spectrophotometry

UV-Vis spectra were taken on a Varian Cary 100 Bio Spectrophotometer with a Peltier 6-cell holder using 1 cm path length using either 1 ml quartz cuvettes or polystyrene disposable cuvettes.

## Bioinformatic Analysis

| | Template | % i.d. | PDB Title |
|---|---|---|---|
| 1 | 6jzu | 26 | Acyl-acyl carrier protein (acyl-ACP) reductase (AAR) in complex with aldehyde deformylating oxygenase (ADO) |
| 2 | 1gpj | 17 | Glutamyl-tRNA reductase from *Methanopyrus kandleri* |
| 3 | 4n7r | 12 | *Arabidopsis* glutamyl-tRNA reductase in complex with its binding protein |
| 4 | 2egg | 14 | Shikimate 5-dehydrogenase (AroE) from *Geobacillus kaustophilus* |
| 5 | 3don | 13 | Shikimate dehydrogenase from *Staphylococcus epidermidis* |
| 6 | 3o8q | 13 | Shikimate 5-dehydrogenase (AroE) from *Vibrio cholerae* |
| 7 | 1nyt | 15 | Shikimate dehydrogenase AroE complexed with $NADP^+$ |
| 8 | 1p74 | 18 | shikimate dehydrogenase (AroE) from *Haemophilus influenzae* |
| 9 | 3pwz | 15 | An AroE-like 1 (Ael1) enzyme from *Pseudomonas putida* |
| 10 | 3pgj | 14 | Shikimate 5-dehydrogenase (AroE) from *Vibrio cholerae* |
| 11 | 4omu | 12 | Shikimate dehydrogenase (AroE) from *Pseudomonas putida* |
| 12 | 1nvt | 14 | Shikimate dehydrogenase (AroE or mj1084) in complex with $NADP^+$ |
| 13 | 2hk8 | 14 | Shikimate dehydrogenase from *Aquifex aeolicus* |

**Table S1**: Closest structures to PigE in the Protein Database (PDB), from Phyre2 [L. A. Kelley *et al.*, *Nature Protocols*, 2015, 10, 845–858].

### BLAST comparison between N-terminal domain of PigE and AAR from *Synechococcus elongatus*

```
NCBI Program Blast 2 sequences
Query ID    Q5W267.1 (amino acid)
Query Descr    RecName: Full=Aminotransferase PigE [Serratia sp. ATCC 39006] ...
Query Length    360
Subject ID    Q54765.1 (amino acid)
Subject Descr    RecName: Full=Long-chain acyl-[acyl-carrier-protein] reductase;
Short=AAR; Short=Acyl-ACP reductase [Synechococcus elongatus PCC 7942 = FACHB-805]
Subject Length    341

Score           Expect   Method           Identities   Positives     Gaps
78.2 bits(191)  1e-20    Compositional    94/355(26%)  155/355(43%)  34/355(9%)
                         matrix adjust.

Query  3     FGFIAHPTSVGLKRYVKMIDLLQRNSTELHSGYKRDLWRRENLVPFMNFAKITSATGATC  62
             FG I H TS+   R     D+ +R   + ++    + W       ++    +TSATG
Sbjct  2     FGLIGHLTSLEQAR-----DVSRRMGYDEYADQGLEFWSSAP-PQIVDEITVTSATGKVI  55

Query  63    EGVIKYM-PLVADEMLADAR--GIANRVVSGIEELVEDGAELVGLGGFTSIVGRRGEATA  119
              G  +Y+      EMLA  R      +V++ +    + G ++  LGGFTSI+    +  +
Sbjct  56    HG--RYIESCFLPEMLAARRFKTATRKVLNAMSHAQKHGIDISALGGFTSIIFENFDLAS  113

Query  120   EKSPVPVT-------SGNSLTTYAGYKALMQIQSWLDIQPEQEPVAIVGYPGSICLALSR  172
              +     T       +GN+ T Y   + +      L I   Q  VA+VG  G I  A+ R
Sbjct  114   LRQVRDTTLEFERFTTGNTHTAYVICRQVEAAAKTLGIDITQATVAVVGATGDIGSAVCR  173
```

```
Query  173  LL-LAQGFSLHLLHRAGHKDEDELLSHLPEQYRSRVTLTSDPEDLYPRCK--LFVAATSA  229
             L L  G    +L      +  D L + L     R ++       E   P      ++VA+
Sbjct  174  WLDLKLGVGDLILTARNQERLDNLQAELG---RGKIL---PLEAALPEADFIVWVASMPQ  227

Query  230  GGVIDPYKLQPGSVFIDVALPRDINSDTRPDRDDILIIDGGCVTATDAV--KLGGESLNV  287
            G VIDP   L+    V ID    P+++ S   +     + I +++GG V       +   ++     +
Sbjct  228  GVVIDPATLKQPCVLIDGGYPKNLGSKVQG--EGIYVLNGGVVEHCFDIDWQIMSAAEMA  285

Query  288  TIKQQLNGCMAETIVLALENRRENFSLGR-YLALDNVLEIGELAEKHGFLVYPLA  341
              ++Q+   C AE ++L   E     NFS GR   + ++ +   IGE + +HGF    PLA
Sbjct  286  RPERQMFACFAEAMLLEFEGWHTNFSWGRNQITIEKMEAIGEASVRHGF--QPLA  338
```

## Alignment of Homology Model for PigE N-terminal domain with AAR (PDB 6jzy)

Alignment was performed in PyMol using the “pair-fit” function. The $\alpha$-carbons of seven pairs of conserved residues in the centre of well conserved regions (shown in red in the above alignment) were chosen for the fitting (including the active site Cys 294 in AAR with Cys 296 in PigE). The R.M.S.D. was 1.105 Å.

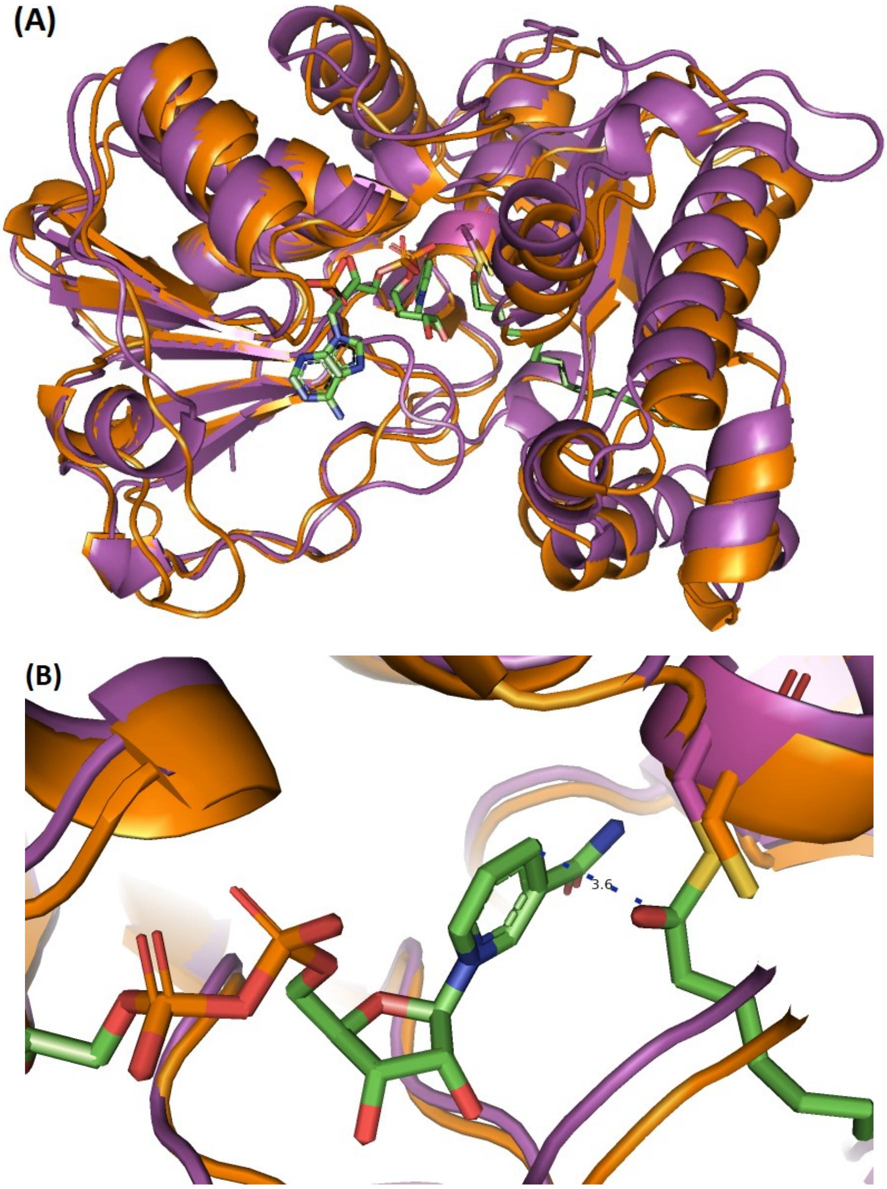

**Fig. S1.** Alignment of Acyl ACP reductase (AAR) from *Synechococcus elongatus* PCC 7942 (PDB 6jzy, magenta) with the homology model generated by Phyre2 for the N-terminal domain of PigE (orange). The protein backbones are shown in cartoon representation and $NADP^+$ and the dodecanoyl group (both with green carbon atoms) and the active site cysteine residues are shown in stick form. (A) complete structure; (B) detail of the active site showing the proximity of C-4 of the nicotinamide ring of $NADP^+$ to the thioester carbon atom (3.6 Å) in AAR. Figures were drawn using Pymol [https://pymol.org/].

## PCR

| Sequence 5'-3' | Restriction enzyme | Usage |
|---|---|---|
| GAGAA**GAGCTC**ATGAAGTTTGGATTTATCGCTC | *SacI* | *pigE* amplification upstream |
| GG**CTGCAG**TTACTCTAAAAATGTTGATAGC | *PstI* | *pigE* amplification downstream |

**Table S1**: Oligonucleotides for PCR (restriction site in bold)

| Compound | Volume (µl) |
|---|---|
| gDNA template | 1 |
| Oligonucleotide 1 (10 µM) | 2.5 |
| Oligonucleotide 2 (10 µM) | 2.5 |
| dNTP (10 mM) | 0.5 |
| 5x HF Buffer | 5 |
| Phusion® Hi-Fidelity DNA polymerase | 0.2 |
| SD-$H_2O$ | Up to 50 µl |

**Table S2:** Composition of PCR mixture

### PCR cycle

PCR cycling parameters included initial denaturation (5 min, 95 °C), then 30 cycles of denaturation (30 s, 95 °C), annealing (30 s, 55 °C) and extension (2 min, 72 °C), and a final extension (10 min, 72 °C). The amplified PCR products were analysed by agarose gel electrophoresis. 0.8% (w/v) agarose gel containing ethidium bromide was run at 85 V in 1×TAE buffer.

### Plasmid construction (pPigE)

PCR amplification was conducted in the conditions described above*. Serratia* sp. 39006 gDNA was amplified using the pair of oligonucleotides in **Table S1**. After purification of the PCR product of the correct size by agarose gel electrophoresis, it was digested with *SacI*/*PstI* restriction enzymes for 1 h at 37 °C and ligated together with a compatibly digested pQE80-L::*oriT.* The generated plasmids were transformed into *E. coli* DH5α and sequenced to confirm the absence of mutation.

| **Strain** | **Genotype/phenotype** | **Source or ref.** |
|---|---|---|
| *Serratia* | | |
| ATCC 39006 | Wild type (Car+, Pig+ ) | Bycroft *et al.*[1] |
| *NW6* (noted *ΔpigE*) | In-frame pigEΔ (10321–12865, 848 aa Δ) | Williamson *et al.*[2] |
| *NW13* (noted *ΔpigD*) | In-frame *pigDΔ* (7895–9446, 517 aa Δ) | Williamson *et al.* |
| *E. coli* | | |
| DH5α | *supE*44, *hsdR17*($r_K^-$ $m_K^-$), *thi-1, recA1, gyrA96* ($Nal^R$), *relA1, Δlac* (*lacIZYA-argF*) *U169 deoR* (φ80 *lacZ*ΔM15) | Grant *et al.*[3] |
| BL21 (DE3) | *B $F^-$ ompT gal dcm lon $hsdS_B$($r_B^-m_B^-$) λ(DE3 [lacI lacUV5-T7p07 ind1 sam7 nin5]) $[malB^+]_{K-12}$($λ^S$)* | Studier *et al.*[4] |
| **Plasmid** | | |
| pQE80-L::*oriT* | 6xHis fusion expression vector containing *oriT* gene for bacterial conjugation, $Amp^R$ | Monson *et al.*[5] |
| pPigE | 2562 bp *PstI*/*SacI* containing N-$His_6$ tag *pigE* ligated into pQE80-L::*oritT* | This study |

**Table S3:** List of bacterial strains and plasmids


1 B. W. Bycroft, C. Maslen, S. J. Box, A. G. Brown, J. W. Tyler, *J. Chem. Soc. Chem. Commun.* **1987**, *0*, 1623.

2 N. R. Williamson, H. T. Simonsen, R. A. A. Ahmed, G. Goldet, H. Slater, L. Woodley, F. J. Leeper, G. P. C. Salmond, *Mol. Microbiol.* **2005**, *56*, 971.

3 S. G. Grant, J. Jessee, F. R. Bloom, D. Hanahan, *Proc. Natl. Acad. Sci.* **1990**, *87*, 4645.

4 F. W. Studier, B. A. Moffatt, *J. Mol. Biol.* **1986**, *189*, 113.

5 R. Monson, D. S. Smith, M. A. Matilla, K. Roberts, E. Richardson, A. Drew, N. Williamson, J. Ramsay, M. Welch, G. P. C. Salmond, *Front. Microbiol.* **2015**, *6*, 1442.

## Protein purification

| | |
|---|---|
| Lysis buffer (pH 8.0) | 50 mM $NaH_2PO_4$ |
| | 300 mM NaCl |
| | 20 mM imidazole |
| Wash buffer (pH 8.0) | 50 mM $NaH_2PO_4$ |
| | 300 mM NaCl |
| | 20 mM imidazole |
| | 20% glycerol (v/v) |
| Elution buffer (pH 8.0) | 50 mM $NaH_2PO_4$ |
| | 300 mM NaCl |
| | 250 mM imidazole |
| Storage buffer (pH 8.0) | 20 mM Tris-HCl (pH 7.0) |
| | 250 mM NaCl |
| | 1 mM EDTA |
| | 1 mM DTT |

**Table S4:** Protein purification buffers

**Preparation of cell lysates**: Cells transformed with suitable vector-constructs were grown at 37 °C on a shaker at 250 rpm to obtain an $OD_{600}$ of 0.6. Cultures were then induced with 1 mM isopropyl β-D-thiogalactopyranoside (IPTG) at 16 °C for 14-16 h. Aliquots were collected before and after the induction by IPTG to monitor the expression of protein and analysed by Sodium Dodecyl Sulphate-Polyacrylamide Gel Electrophoresis (SDS-PAGE). Cells were harvested by centrifuging at 5000 rpm for 20 min at 4 °C. The cell pellet was resuspended in the lysis buffer including complete-mini-EDTA-free protease inhibitor cocktail (1 tablet per 10 ml lysis buffer) from Roche.

**For protein purification from soluble fraction**: The above lysis buffer was supplemented with 1% Triton-X100. The ice-water jacketed suspension was sonicated and then centrifuged at 10,000 rpm at 4 °C for 30 min. The clarified lysate was loaded onto a Ni- NTA ($Ni^{2+}$-nitrilotriacetate) column (Qiagen) for affinity purification. The column was washed twice with wash buffer and the bound protein was eluted (1 ml fractions) with elution buffer and the fractions analysed by SDS-PAGE. The fractions containing purified protein were pooled and dialyzed in storage buffer.

## Characterisation of PigE

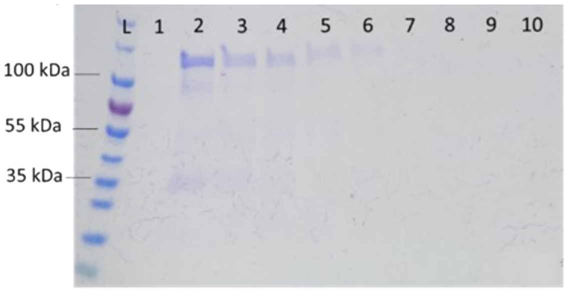


**Figure S2:** Analysis of PigE by SDS-PAGE electrophoresis

**Analysis of extract from S39006 ΔpigD cultured with thioester 13 and of extracts from S39006 WT**

**HPLC conditions.** HPLC analysis were performed on an Agilent 1100 Series system fitted with an autosampler and a UV/Vis detector. Eluents were supplemented with 0.1% formic acid. Extracts were run with a Phenomenex Jupiter 5μ C18 300A with an acetonitrile gradient (5 to 100% in 30.5 min, then back to 5% in 30 s and constant 5% for 9 minutes). The absorbances at 500 and 535 nm were monitored.

**LC-MS analysis of the HPLC peaks.** The analysis was performed on extracts from S39006 WT

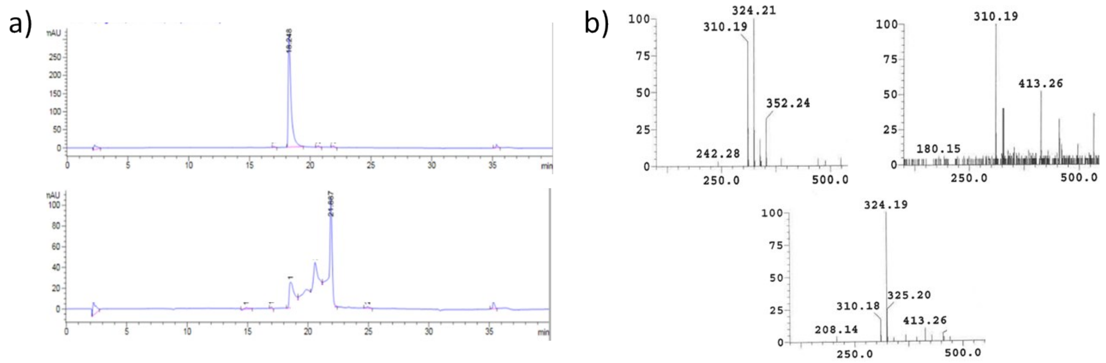


**Figure S4: Analysis of the prodigiosin extract** a) HPLC trace of the two peaks after separation by HPLC (top, peak at 17.3 min, bottom, peak at 21.3 min, detection at 535 nm); b) MS analysis, top left: crude extract, top right, peak at 17.3 min, bottom, peak at 21.3 min.

**Determination of the amino-donor in the aminotransferase activity of PigE**

20 µg of PigE in Tris-HCl buffer (510 mM, pH=8.0) was incubated with 5 mM of amino acid (total volume = 1 ml) at 30 °C. A UV/Vis spectra was recorded after 0 h, 1 h, 24 h, and, in some instances, 48 h. To compensate for the absorbance of the starting materials, the absorbance at 0 h was subtracted from the subsequent measurements.

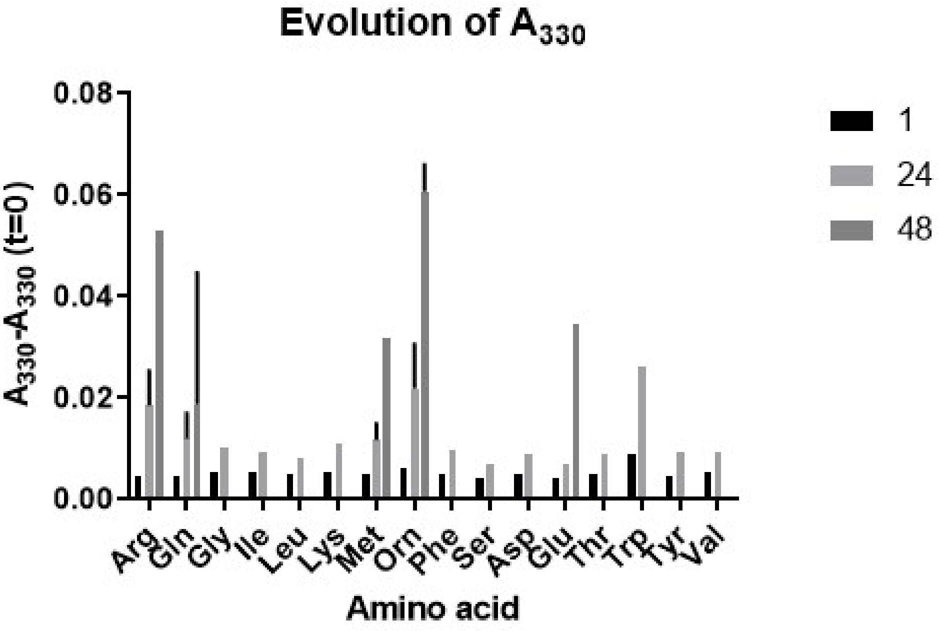


**Figure S5:** Evolution of the $A_{330}$ of purified PigE in presence of a variety of amino acids.

## Sample preparation for mass spectrometry

Eppendorf tubes were charged with 1 mM of thioester **13**, 1.25 mM of ornithine and PLP, and 1.25 mM of NADH or NADPH. For the negative control, no NAD(P)H was added. Cell lysate containing PigE or the corresponding control was added. The resulting 0.5 ml were gently shaken overnight at 30 °C. The insoluble fraction was eliminated by centrifugation and the soluble fraction analysed directly by LCMS.

## Synthesis

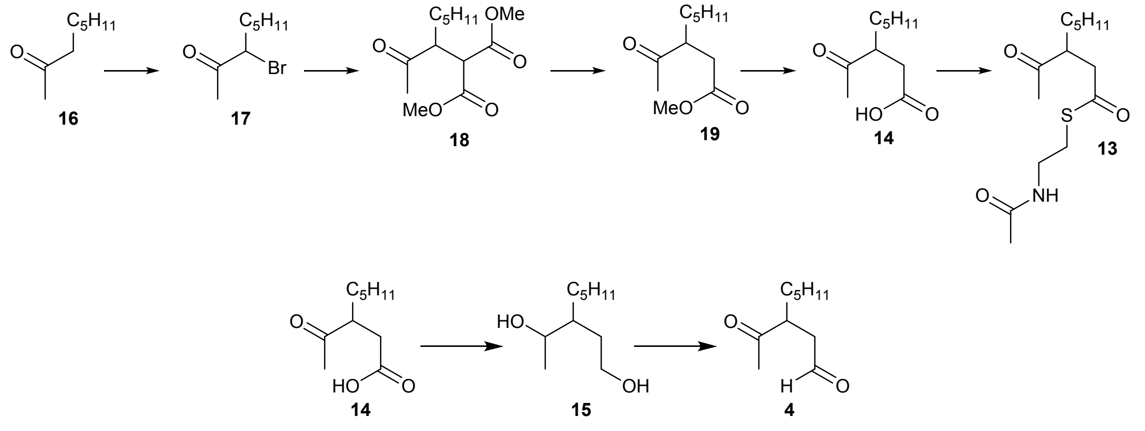


*3-Bromooctan-2-one* **17**

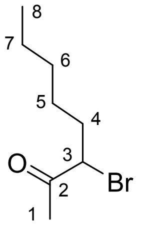

To a mixture of 2-octanone **16** (5 g, 39 mmol) and $CHCl_3$ (40 ml) was added $NH_4OAc$ (300 mg), followed by N-bromosuccinimide (7 g, 39 mmol). The mixture was heated at 80 °C and stirred for 24 h. Hexane (40 ml) was then added and the mixture was filtered. The filtrate was washed with sat. aq. $NaHCO_3$ and brine. The organic layer was dried ($MgSO_4$) and the solvent was removed under reduced pressure. Purification by flash column chromatography (EtOAc/hexane 1:9) gave a 9:1 mixture of **17** and 1-bromooctanone **17b** (1.285 g, 6.2 mmol, 16%). Pure **17** was obtained by a kinetic separation as follows. The mixture of **17** and **17b** (1 g, 4.8 mmol) was dissolved in acetone (5 ml) and $K_2CO_3$ (0.5 g, 3.6 mmol) and dimethyl malonate (300 µl, 2 mmol, 0.4 eq.) were added. The mixture was stirred at 60 °C for 2 h. $CH_2Cl_2$ (15 ml) was added and the mixture was washed with brine. The organic layer was dried ($MgSO_4$), concentrated under reduced pressure and purified by flash column chromatography, giving pure **17** (846 mg, 4 mmol, 90% recovery) as **17b** reacts preferentially with the enolate of dimethyl malonate.

$^1$H NMR (400 MHz, $CDCl_3$): δ 0.90 (3H, t, *J* 7 Hz, $H_8$), 1.27-1.40 (5H, m, $H_7$,$H_6$, $H_{5a}$), 1.5 (1H, m, $H_{5b}$), 1.85-2.08 (2H, m, $H_4$), 2.37 (3H, s, $H_1$), 4.24 (1 H, t, *J* 7 Hz, $H_3$).

$^{13}$C NMR (100 MHz, $CDCl_3$, HMQC and HMBC used for assignments): 13.9 ($C_8$), 22.4 ($C_7$), 26.0 ($C_1$), 26.9 ($C_5$), 31.1 ($C_6$), 33.5 ($C_4$), 54.4 ($C_3$), 202.19 ($C_2$).

IR (neat): $\nu_{max}$ 2929m, 2860m, 1716s (C=O).

LC-MS: *m/z* calcd for $C_8H_{15}BrO+H^+$: 209.04; found: 209.08.

*Dimethyl 2-(2-oxooctan-3-yl) malonate* **18**

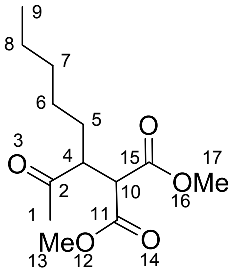

Dimethyl malonate (487 µl, 4.2 mmol) and $K_2CO_3$ (750 mg, 13.4 mmol) were added to **17** (1.5 g, 7.2 mmol) in acetone (7.5 ml). The mixture was stirred at 80 °C overnight. $CH_2Cl_2$ (20 ml) was added and the organic phase was washed with $H_2O$ and brine. The organic phase was dried

($MgSO_4$) and the solvent removed under reduced pressure. Purification by flash column chromatography (hexane/EtOAc) gave **18** (755 mg, 2.9 mmol, 40%).
$^1$H NMR (400 MHz, $CDCl_3$): δ 0.87 (3 H, t, *J* 6 Hz, $H_9$), 1.2-1.34 (6H, m, $H_6$, $H_7$, $H_8$), 1.52 (2H, m, $H_5$), 2.28 (3H, s, $H_1$), 3.34 (1H, dt, *J* 11 & 6 Hz, $H_4$), 3.69 (3H, s, $H_{13}$ or $H_{17}$), 3.75 (3H, s, $H_{13}$ or $H_{17}$), 3.83 (1H, d, *J* 11 Hz, $H_{10}$).
$^{13}$C NMR (100 MHz, $CDCl_3$, HMQC and HMBC used for assignments): δ 13.9 ($C_9$), 22.8 ($C_8$), 25.6 ($C_6$), 29.0 ($C_5$), 30.5 ($C_1$), 31.8 ($C_7$), 50.6 ($C_4$), 52.7 ($C_{13}$ and $C_{17}$), 53.1 ($C_{10}$), 169 ($C_{11}$ and $C_{15}$), 209.9 ($C_2$).
IR (neat): $\nu_{max}$ 2955w, 2862w, 1735s (C=O), 1713 (C=O), 1265m, 1226m, 1151s (C-O).
LC-MS: *m/z* calcd for $C_{13}H_{22}O_5$+$Na^+$: 281.1359; found: 281.1370.

*Methyl 3-acetyloctanoate* **19**

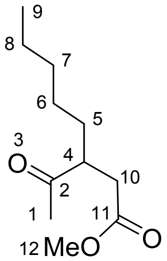


Dimethyl ester **18** (191 mg, 0.74 mmol), NaCl (120 mg, 2 mmol, 2.7 eq.), and $H_2O$ (54 μl, 3 mmol, 4 eq.) in DMSO (12 ml) were stirred at 160 °C under a condenser for 16 h. The mixture was diluted with EtOAc (20 ml) and washed with water and brine. The aqueous phase was reextracted with EtOAc. The organic layers were dried ($MgSO_4$) and evaporated under reduced pressure, giving **19** (110 mg, 0.55 mmol, 74%).
$^1$H NMR (400 MHz, $CDCl_3$): δ 0.88 (3H, t, *J* 12 Hz, $H_9$), 1.26 (6H, m, $H_6$, $H_7$ and $H_8$), 1.41 (1H, m, $H_{5a}$), 1.60 (1H, m, $H_{5b}$), 2.23 (3H, s, $H_1$), 2.35 (1H, dd, *J* 17 & 4 Hz, $H_{10a}$), 2.73 (1H, dd, *J* 17 & 10 Hz, $H_{10b}$), 2.98 (1H, m, $H_4$), 3.64 (3H, s, $H_{12}$).
$^{13}$C NMR (100 MHz, $CDCl_3$) δ 14.0 ($C_9$), 22.4 ($C_8$), 26.6 ($C_6$), 29.5 ($C_1$), 31.3 ($C_5$), 31.7 ($C_7$), 34.9 ($C_{10}$), 47.9 ($C_4$), 51.7 ($C_{12}$), 173.0 ($C_{11}$), 211.1 ($C_2$).

*3-Acetyloctanoic acid* **14**

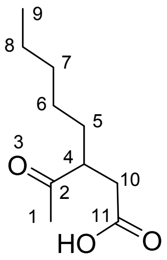


Methyl ester **19** (110 mg, 0.55 mmol) was dissolved in MeOH/$H_2O$ 4:1 (4 ml) and KOH (270 mg, 4.8 mmol) was added. The mixture was heated to 60 °C and stirred for 4 h. Hydrochloric acid 1 M was added slowly until pH<2. The mixture was extracted with $CH_2Cl_2$. Analysis of the organic phase showed presence of DMSO from the previous step and unreacted **19** (<10%). $CH_2Cl_2$ was removed under reduced pressure and the mixture dissolved in $NaHCO_3$ sat. and washed with $CH_2Cl_2$. The aqueous phase was then acidified by adding hydrochloric acid (1 M) and extracted with $CH_2Cl_2$. The organic extract was then dried, and the solvent removed under reduced pressure to give **14** (100 mg, 0.53 mmol, 96%).
$^1$H NMR (400 MHz, $CDCl_3$): δ 0.88 (3H, t, *J* 7 Hz, $H_9$), 1.23-1.34 (6H, m, $H_6$, $H_7$ and $H_8$), 1.42 (1H, m, $H_{5a}$) 1.59 (1H, m, $H_{5b}$), 2.20 (3H, s, $H_1$), 2.39 (1H, dd, *J* 17 & 4 Hz, $H_{10a}$), 2.76 (1H, dd, *J* 17 & 10 Hz, $H_{10b}$), 2.93 (1H, m, $H_4$).
$^{13}$C NMR (100 MHz, $CDCl_3$, HMQC used for assignments): δ 14.11 ($C_9$), 22.57 ($C_8$), 26.69 ($C_7$), 29.41 ($C_1$), 31.27 ($C_6$), 31.89 ($C_5$), 35.09 ($C_{10}$), 47.94 ($C_4$), 177.55 ($C_{11}$), 210.74 ($C_2$);
IR (neat): $\nu_{max}$ 2958m, 2928m, 2861m, 1707s (C=O), 1374 (C-O carboxylic acid);
UV Absorbance: $\lambda_{max}$ 276 nm.
LC/MS *m/z* calcd for $C_{10}H_{18}O_3$-$H^+$: 185.12; found: 185.1.

*S-(2-Acetamidoethyl) 3-acetyloctanethioate* **13**

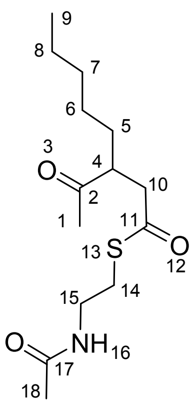


Carboxylic acid **14** (50 mg, 0.27 mmol) was dissolved in $CH_2Cl_2$ (0.5 ml) and cooled on ice. EDC (57 mg, 0.36 mmol) and DMAP (6 mg, 0.05 mmol) were added and the mixture was stirred on ice for 15 min. N-Acetylcysteamine (36 mg, 0.3 mmol, 1.1 eq.) was added and the mixture was allowed to reach r.t. and was stirred overnight. The solvent was removed under reduced pressure and the residue dissolved in $CHCl_3$ (ca. 2 ml) and washed with hydrochloric

acid (0.1 M) and brine. The aqueous layers were reextracted with $CHCl_3$. The organic layers were combined and dried ($MgSO_4$). The solvent was removed under reduced pressure. Purification by flash column chromatography ($CH_2Cl_2$/MeOH 9:1) gave **13** (13 mg, 16%).
$^1$H NMR (400 MHz, $CDCl_3$): δ 0.88 (3H, t, *J* 7 Hz, $H_9$), 1.27 (6H, m, $H_6$, $H_7$ and $H_8$), 1.43 (2H, m, $H_5$), 1.96 (3H, s, $H_{18}$), 2.21 (3H, s, $H_1$), 2.63 (1H, m, $H_{10a}$), 3.04 (4H, m, $H_4$, $H_{10b}$ and $H_{14}$), 3.42 (2H, m, $H_{15}$), 5.82 (1H, s, NH).
$^{13}$C NMR (100 MHz, $CDCl_3$, HMQC and HMBC used for assignments): δ 14.09 ($C_9$), 22.54 ($C_8$), 23.30 ($C_{18}$), 26.64 ($C_6$), 28.77 ($C_{14}$), 29.39 ($C_1$), 31.22 ($C_5$), 31.87 ($C_7$), 39.61 ($C_{15}$), 44.69 ($C_{10}$), 48.56 ($C_4$), 170.51 ($C_{17}$), 198.72 ($C_{11}$), 210.12 ($C_2$).
IR (neat): $\nu_{max}$ 2948m, 2932m, 2854m, 1709s (C=O ketone), 1687s and 1657s (C=O amide and thioester), 1541m (NH bend).
LC-MS: *m/z* calcd for $C_{14}H_{25}NO_3S+Na^+$: 310,1447; found: 310,1456.

*3-Pentylpentane-1,4-diol* **15**

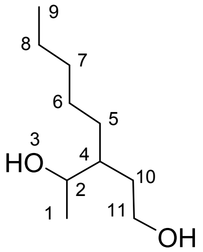


$LiAlH_4$ (1 M in THF, 2.1 ml, 2.1 mmol) was cooled to 0 °C. Acid **14** (100 mg, 0.53 mmol) in THF (1.25 ml) was added dropwise and the mixture was then allowed to warm to r.t. and stirred overnight. $H_2O$ (75 µl), NaOH (15% in $H_2O$, 75 µl) and $H_2O$ (225 µl) were then added slowly at 0 °C. The cold bath was removed, and the mixture was stirred for a further 1 h. The mixture was diluted with THF, dried ($MgSO_4$) and concentrated under reduced pressure to give **15** (80 mg, 0.46 mmol, 87%) as a mixture of diastereoisomers.
$^1$H NMR (400 MHz, $CDCl_3$): δ 0.88 (3H, t, *J* 7 Hz, $H_9$), 1.15 and 1.21 (3H, d, *J* 6 Hz, $H_1$), 1.23-1.35 (8H, m, $H_5$, $H_6$, $H_7$ and $H_8$), 1.46 (1/2H, m, $H_{4a}$), 1.5-1.58 (1-2H, m, $H_{10a1}$), 1.6-1.68 (1 H, m, $H_{10a2}$ and $H_{4b}$), 1.74-1.79 (1H, m, $H_{10b}$), 3.65 (1H, m, $H_{11a}$), 3.77 (1-2H, m, $H_{11b}$, $H_{2a}$), 3.9 (m, 1-2H, $H_{2b}$).
$^{13}$C NMR (100 MHz, $CDCl_3$) δ 14.1 ($C_9$), 18.5 and 21.3 ($C_1$), 22.62 ($C_8$), 26.9 and 27.4 ($C_5$), 30.8 and 31.0 ($C_6$), 32.1 and 32.2 ($C_7$), 32.4 and 32.6 ($C_{10}$), 43.2 and 43.3 ($C_4$), 60.6 and 61.7 ($C_{11}$), 70.1 ($C_2$).
IR (neat): $\nu_{max}$ 3306br (O-H), 2925s, 2857s, 1052s (C-O).

*3-Acetyloctanal* **4**

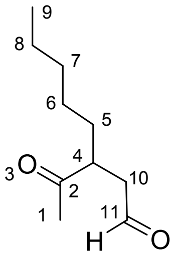


Oxalyl chloride (675 µl, 7.8 mmol) was added dropwise to a stirred solution of DMSO (755 µl, 10.6 mmol) in $CH_2Cl_2$ (10 ml) at -78°C. The mixture was stirred for 10 min at -78°C and then a solution of diol **15** (300 mg, 1.7 mmol) in $CH_2Cl_2$ (5 ml) was added dropwise. The mixture was stirred a further 10 min at -78 °C. $Et_3N$ was then added and the reaction was stirred at r.t. for 30 min. The mixture was diluted with $Et_2O$ (20 ml) and washed with brine. The organic layer was dried ($MgSO_4$) and concentrated under reduced pressure. Purification by flash column chromatography gave **5** (250 mg, 1.5 mmol, 88%).
$^1$H NMR (400 MHz, $CDCl_3$): δ 0.87 (3H, t, *J* 7 Hz, $H_9$), 1.22 (6H, m, $H_6$, $H_7$ and $H_8$), 1.42 (1H, m, $H_{5a}$), 1.58(1H, m, $H_{5b}$), 2.20 (3H, s, $H_1$), 2.45 (1H, dd, *J* 4 & 18 Hz, $H_{10a}$), 2.92 (1H, dd, *J* 10 & 18 Hz, $H_{10b}$), 3.02 (1H, m, $H_4$), 9.70 (1H, s, $H_{13}$).
$^{13}$C NMR (100 MHz, $CDCl_3$, HSQC and HMBC used for assignments): δ 14.0 ($C_9$), 22.4 ($C_8$), 26.6 ($C_6$), 29.4 ($C_1$), 31.2 ( $C_5$), 31.7 ($C_7$), 44.9 ($C_{10}$), 45.9 ($C_4$), 200.7 ($C_{11}$), 210.7 ($C_2$).
IR (neat): $\nu_{max}$ 2957m, 2958m, 2857m, 2750w (aldehyde C-H), 1713 (C=O).
LC-MS: *m/z* calcd for $C_{10}H_{18}O_2+H^+$: 171.1380; found: 171.1379

*3-Bromooctan-2-one* **17**

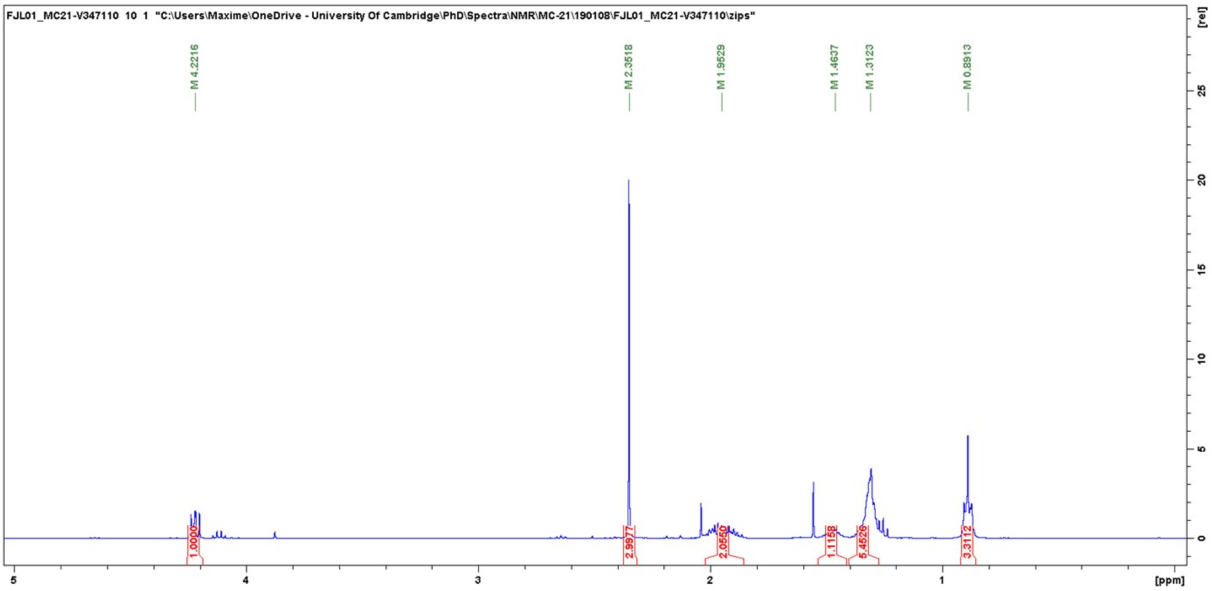


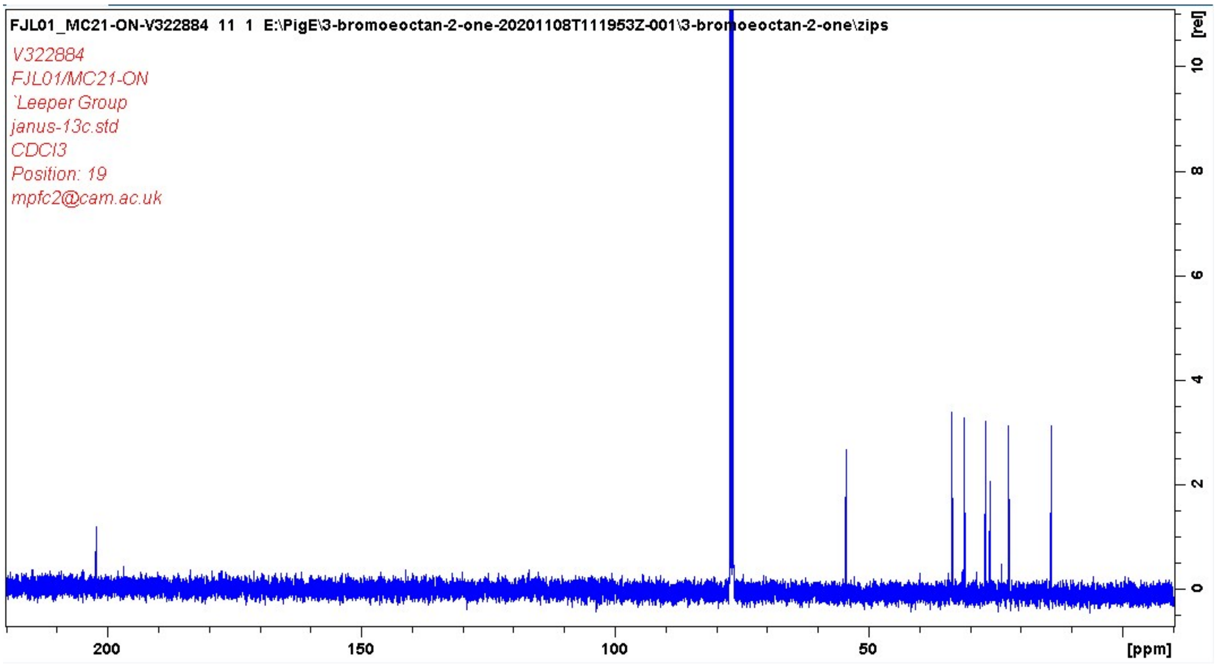

*Dimethyl 2-(2-oxooctan-3-yl) malonate* **18**

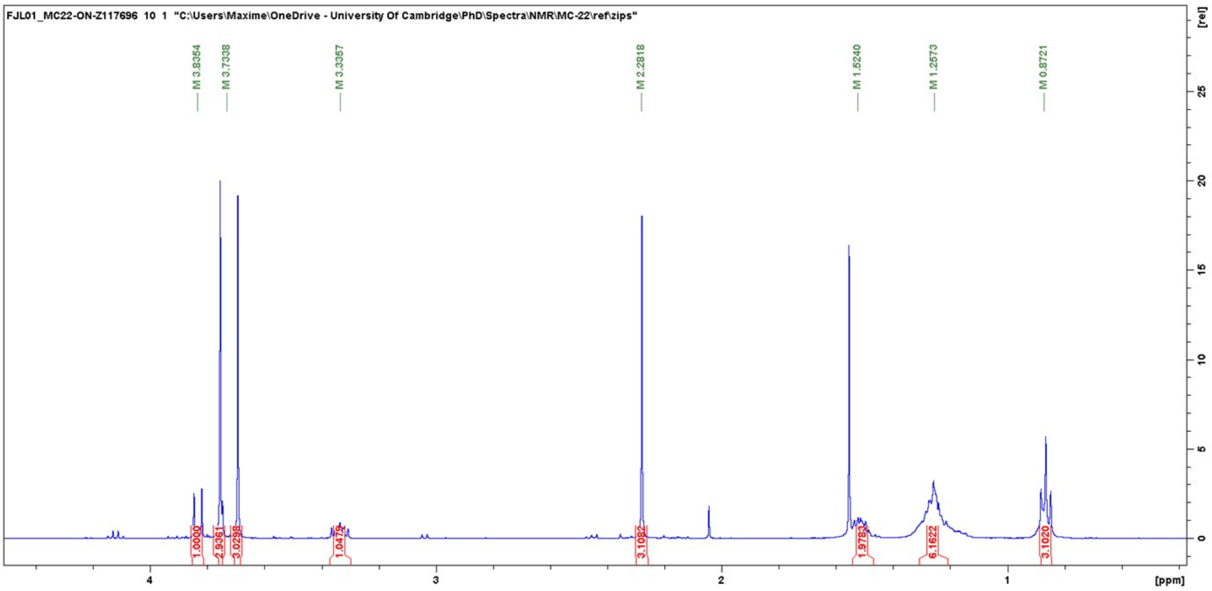


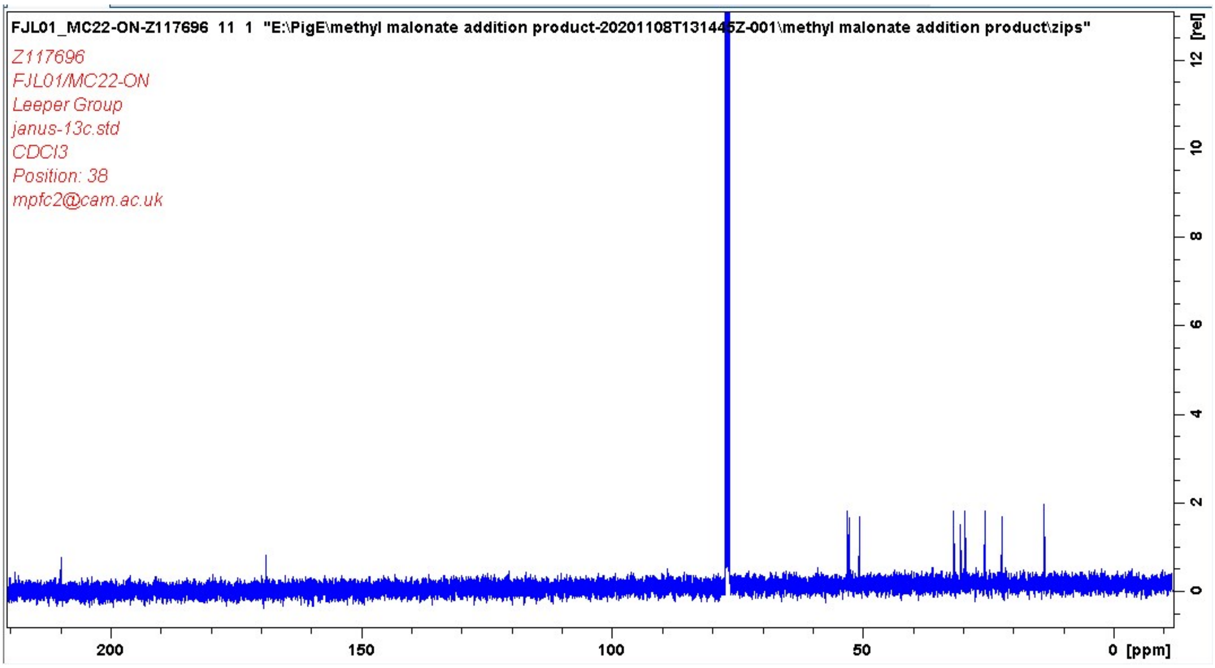

*Methyl 3-acetyloctanoate* **19**

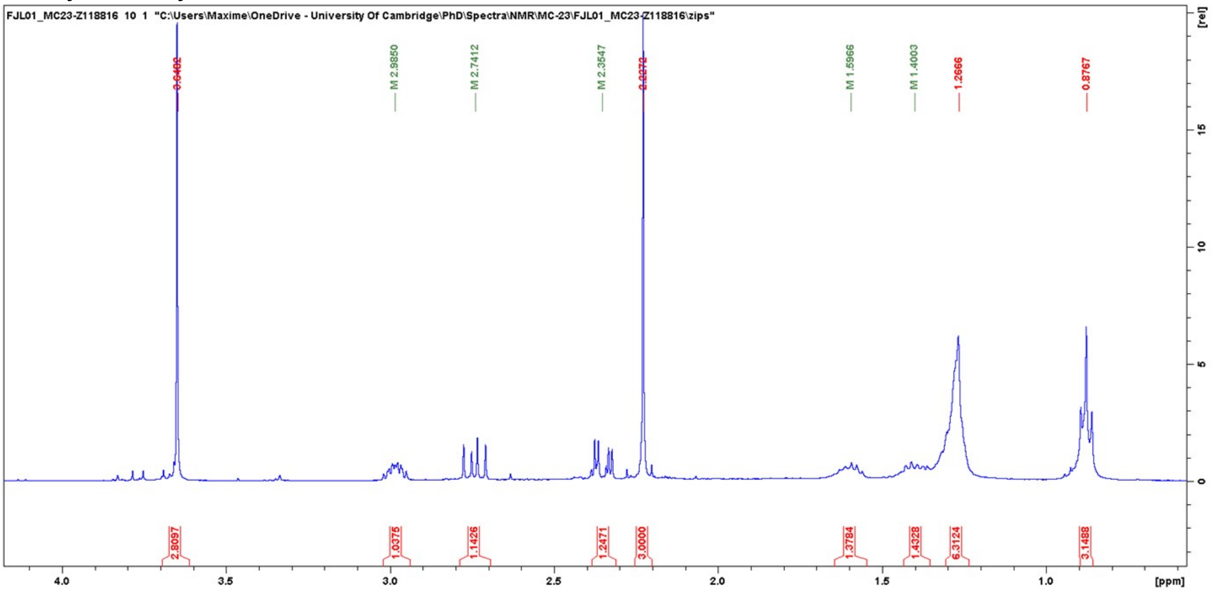


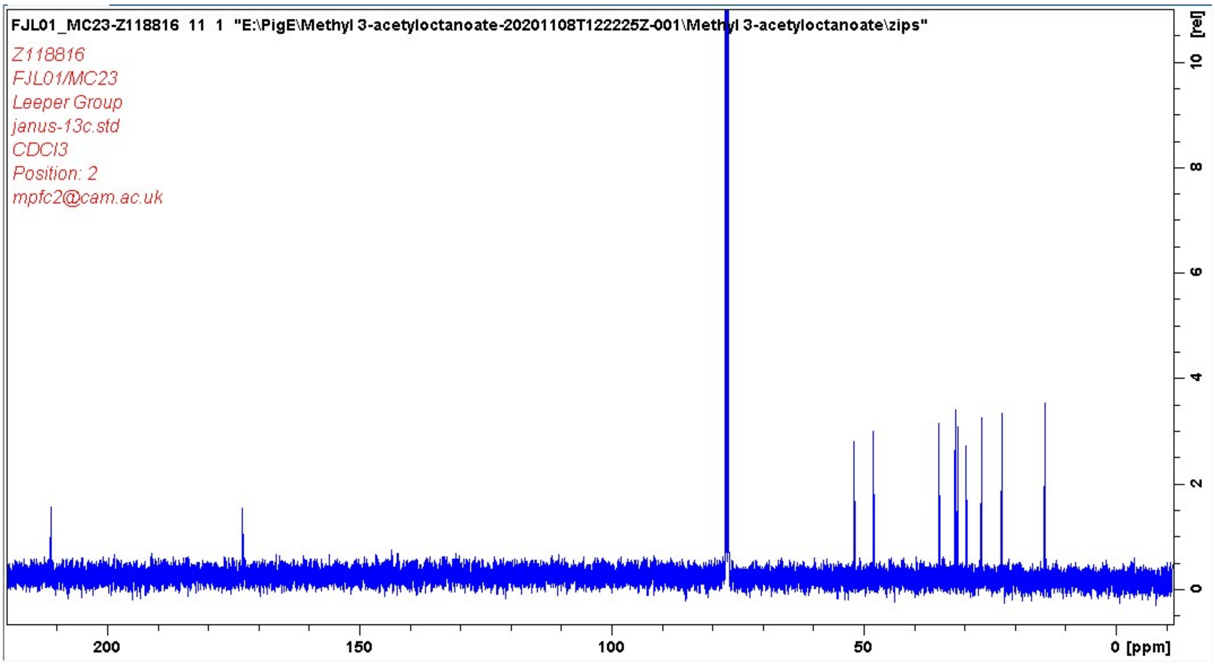

*3-Acetyloctanoic acid* **14**

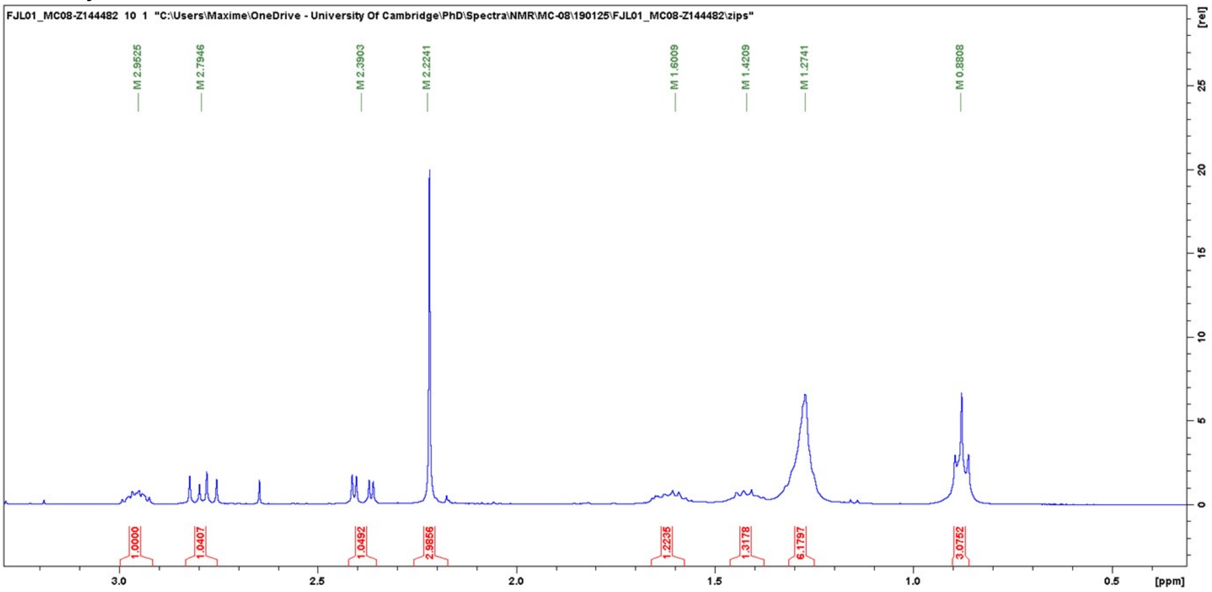


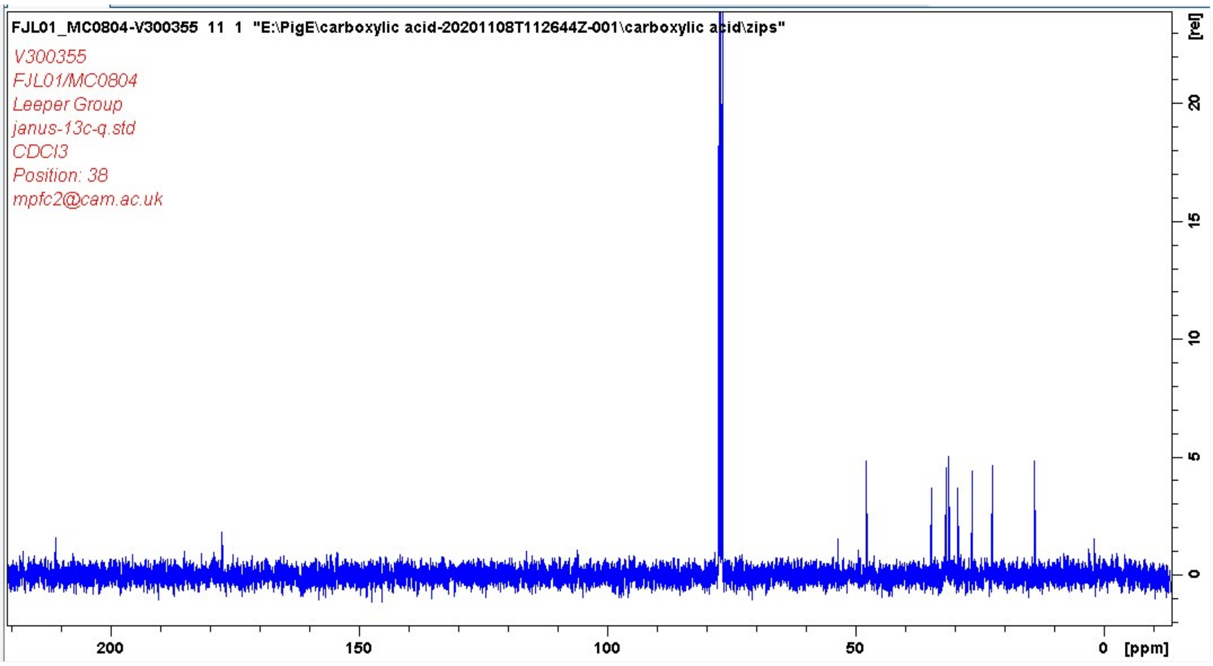

*S-(2-Acetamidoethyl) 3-acetyloctanethioate* **13**

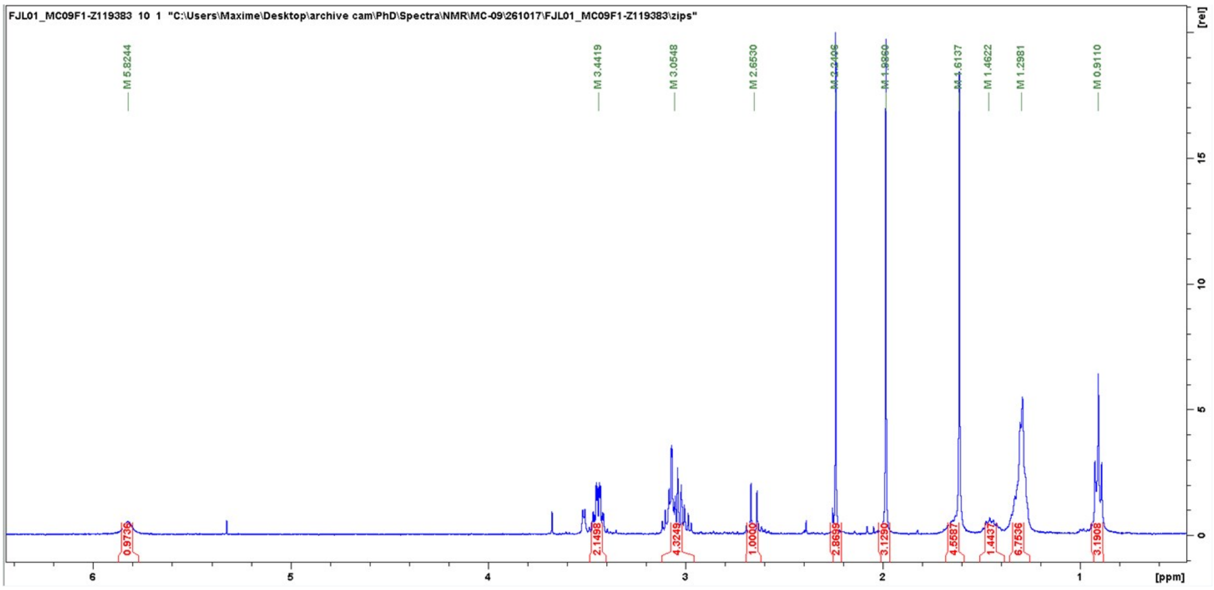


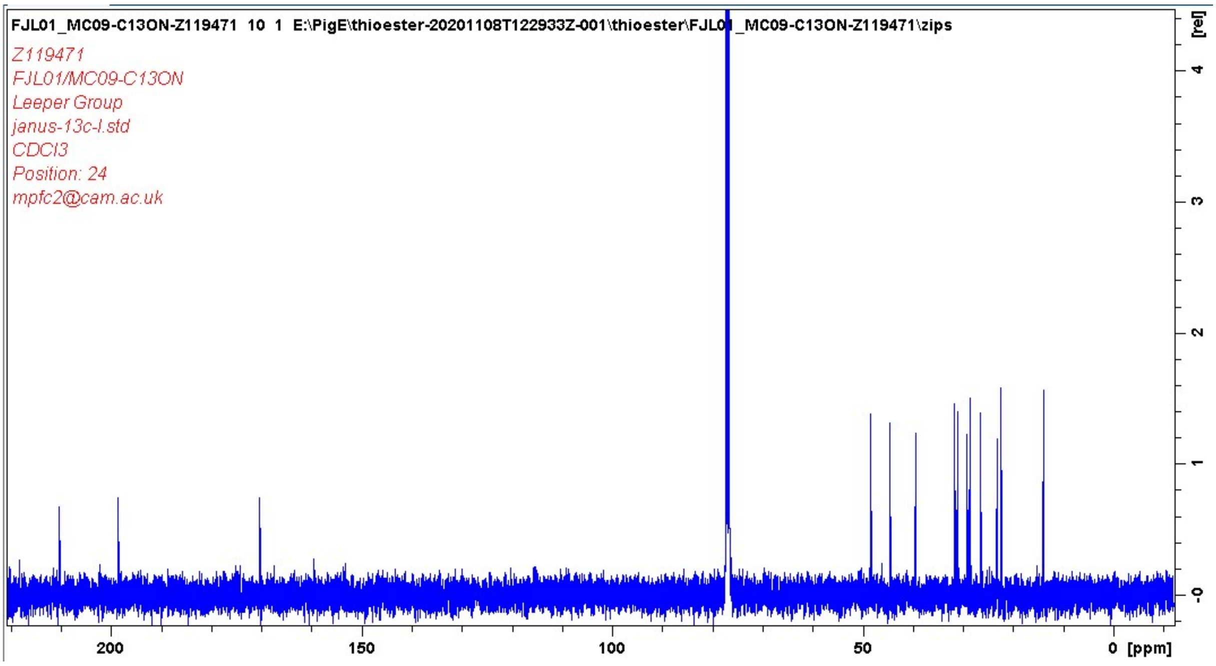

*3-Pentylpentane-1,4-diol* **15** (mixture of diastereoisomers)

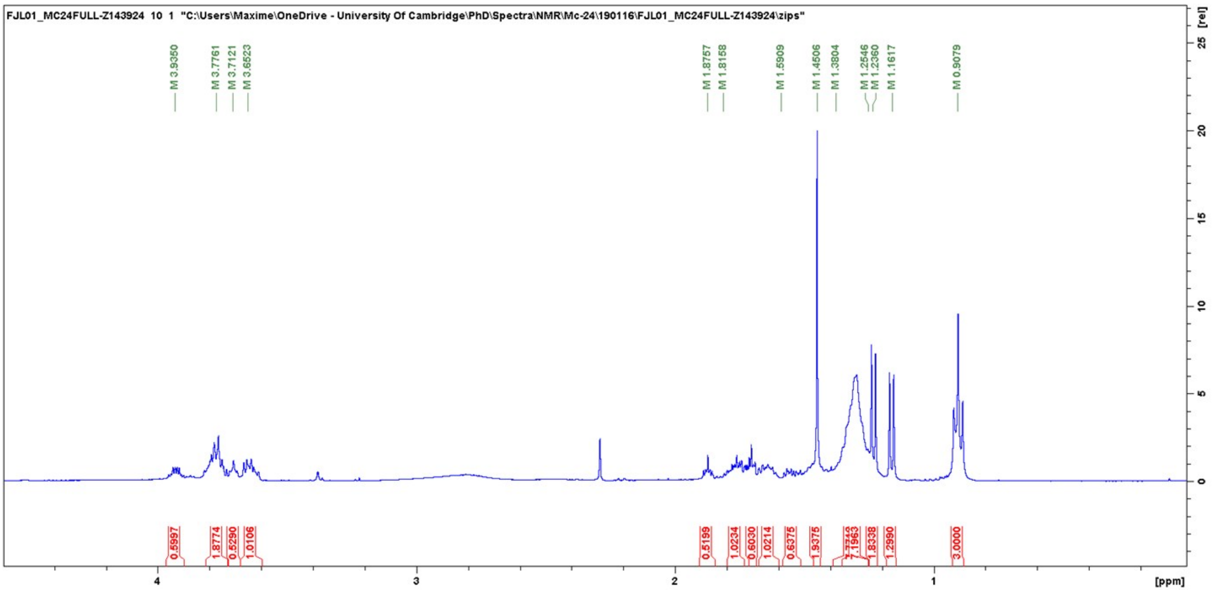


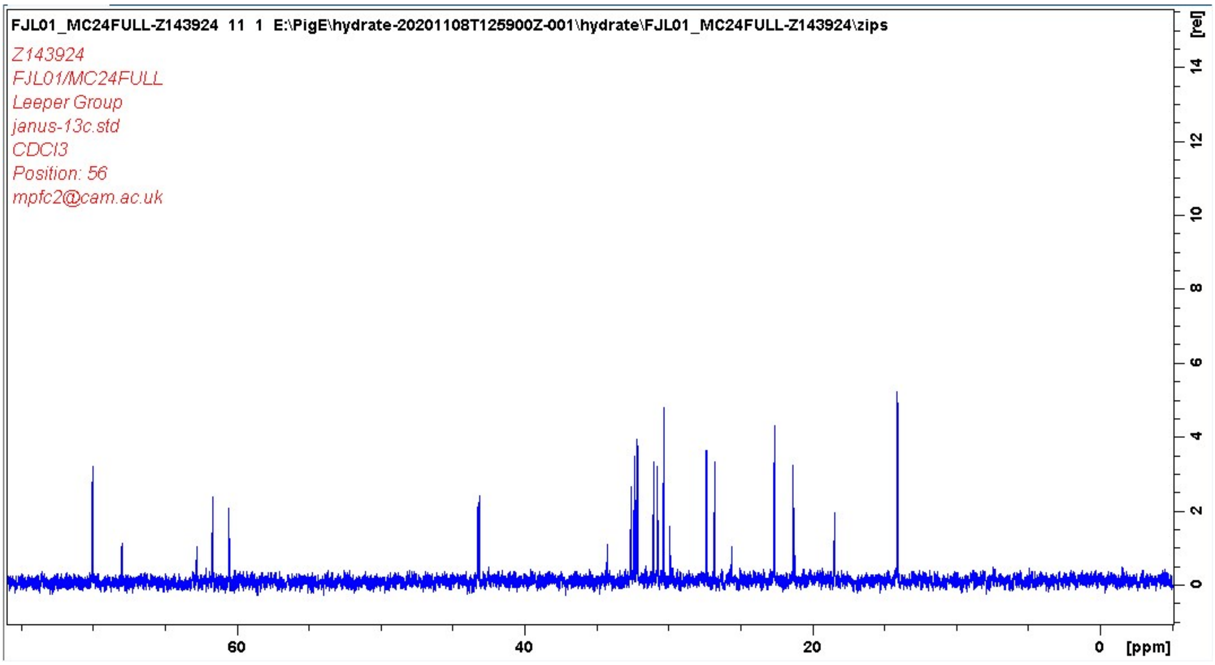

*3-Acetyloctanal* **4**

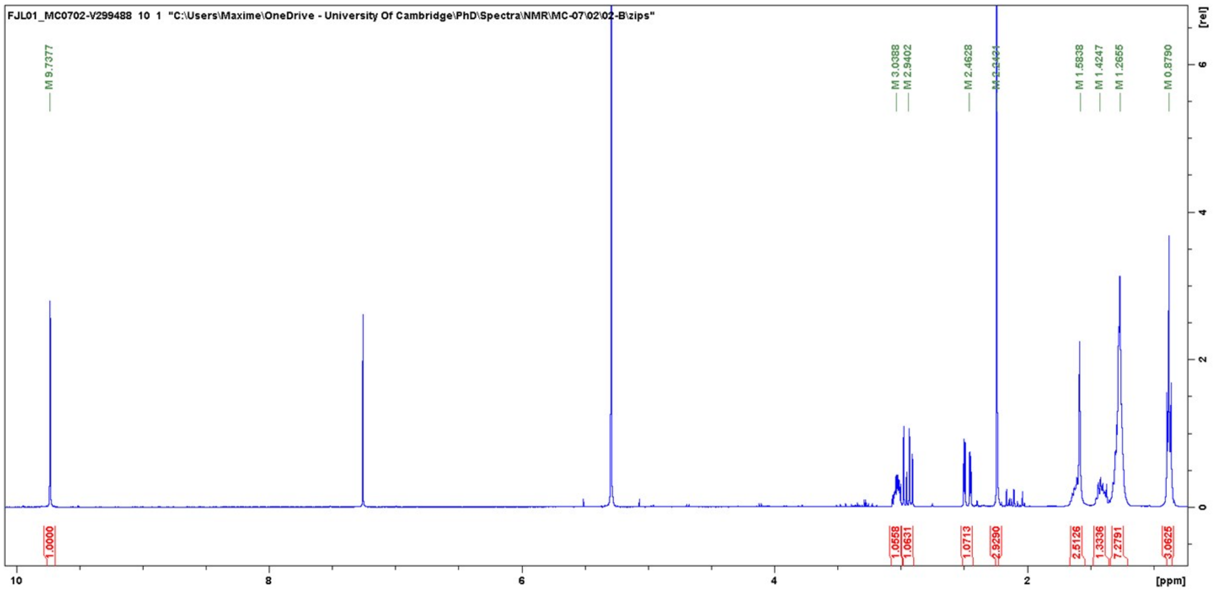


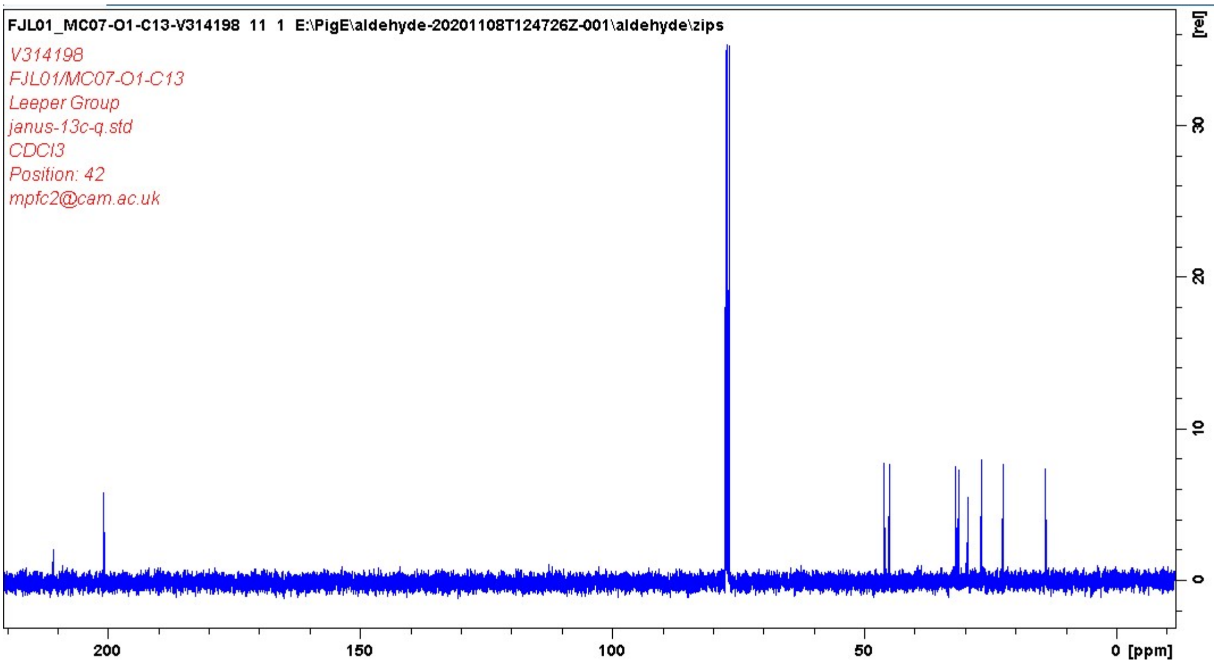

Supplement: CB-002-D0CB00173B-s001 [file CB-002-D0CB00173B-s001.pdf]
